# Supplementary material for: A Humanized Yeast Phenomic Model of Deoxycytidine Kinase to Predict Genetic Buffering of Nucleoside Analog Cytotoxicity
Source: Genes (Basel). 2019 Sep 30;10(10):770. doi: 10.3390/genes10100770 (PMC6826991; doi:10.3390/genes10100770)
Supplement: Supplementary file 1 [file genes-10-00770-s001.zip › Additional_Files/Additional_File7_GOTermHeatmaps.zip!/Additional_File7_GOTermHeatmaps/protein_localization_to_vacuole.pdf]

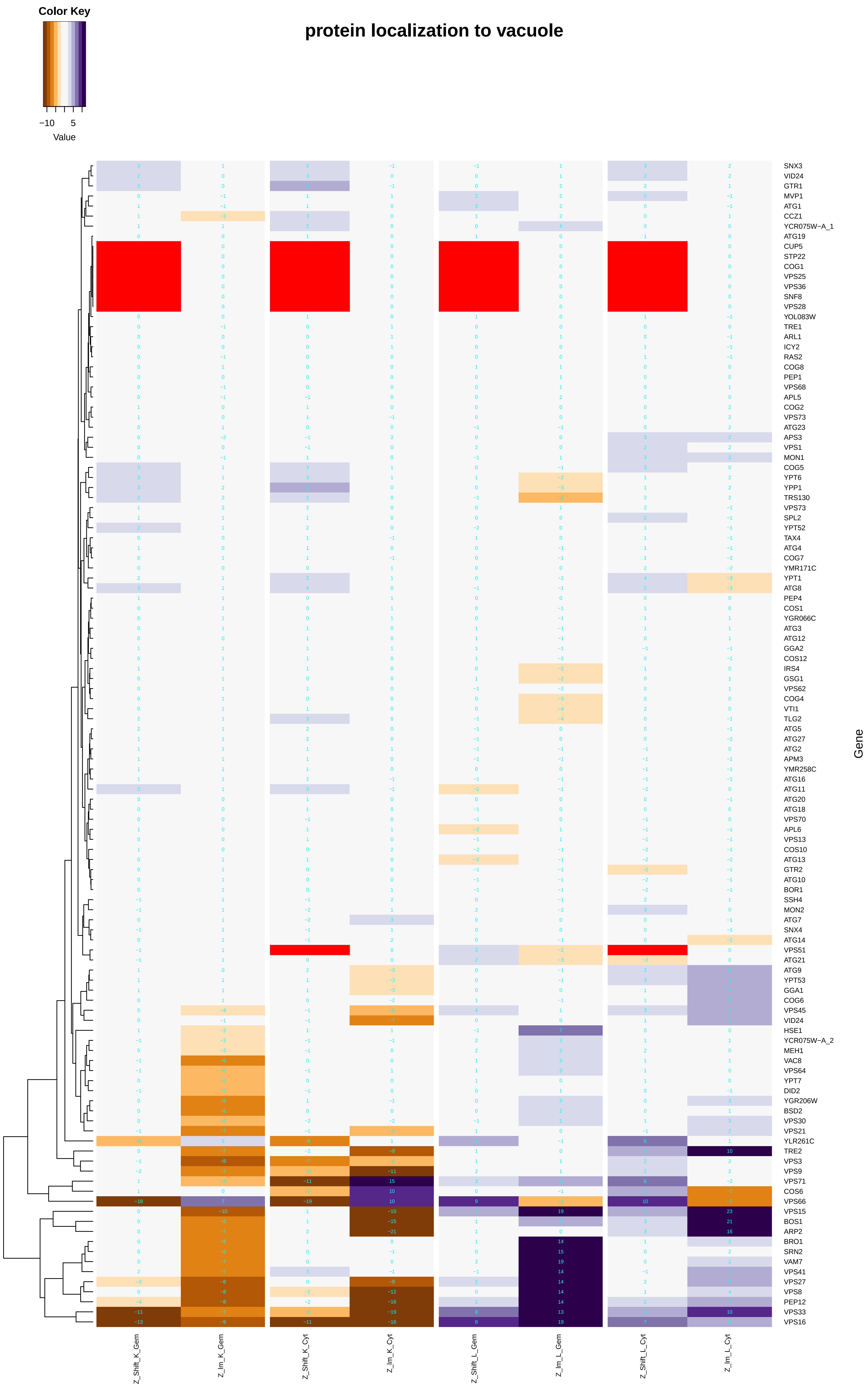
protein localization to vacuole
Color Key
−10 5
Value
Gene
Z_Shift_K_Gem
Z_Im_K_Gem
Z_Shift_K_Cyt
Z_Im_K_Cyt
Z_Shift_L_Gem
Z_Im_L_Gem
Z_Shift_L_Cyt
Z_Im_L_Cyt
SNX3
VID24
GTR1
MVP1
ATG1
CCZ1
YCR075W−A_1
ATG19
CUP5
STP22
COG1
VPS25
VPS36
SNF8
VPS28
YOL083W
TRE1
ARL1
ICY2
RAS2
COG8
PEP1
VPS68
APL5
COG2
VPS73
ATG23
APS3
VPS1
MON1
COG5
YPT6
YPP1
TRS130
VPS73
SPL2
YPT52
TAX4
ATG4
COG7
YMR171C
YPT1
ATG8
PEP4
COS1
YGR066C
ATG3
ATG12
GGA2
COS12
IRS4
GSG1
VPS62
COG4
VTI1
TLG2
ATG5
ATG27
ATG2
APM3
YMR258C
ATG16
ATG11
ATG20
ATG18
VPS70
APL6
VPS13
COS10
ATG13
GTR2
ATG10
BOR1
SSH4
MON2
ATG7
SNX4
ATG14
VPS51
ATG21
ATG9
YPT53
GGA1
COG6
VPS45
VID24
HSE1
YCR075W−A_2
MEH1
VAC8
VPS64
YPT7
DID2
YGR206W
BSD2
VPS30
VPS21
YLR261C
TRE2
VPS3
VPS9
VPS71
COS6
VPS66
VPS15
BOS1
ARP2
BRO1
SRN2
VAM7
VPS41
VPS27
VPS8
PEP12
VPS33
VPS16

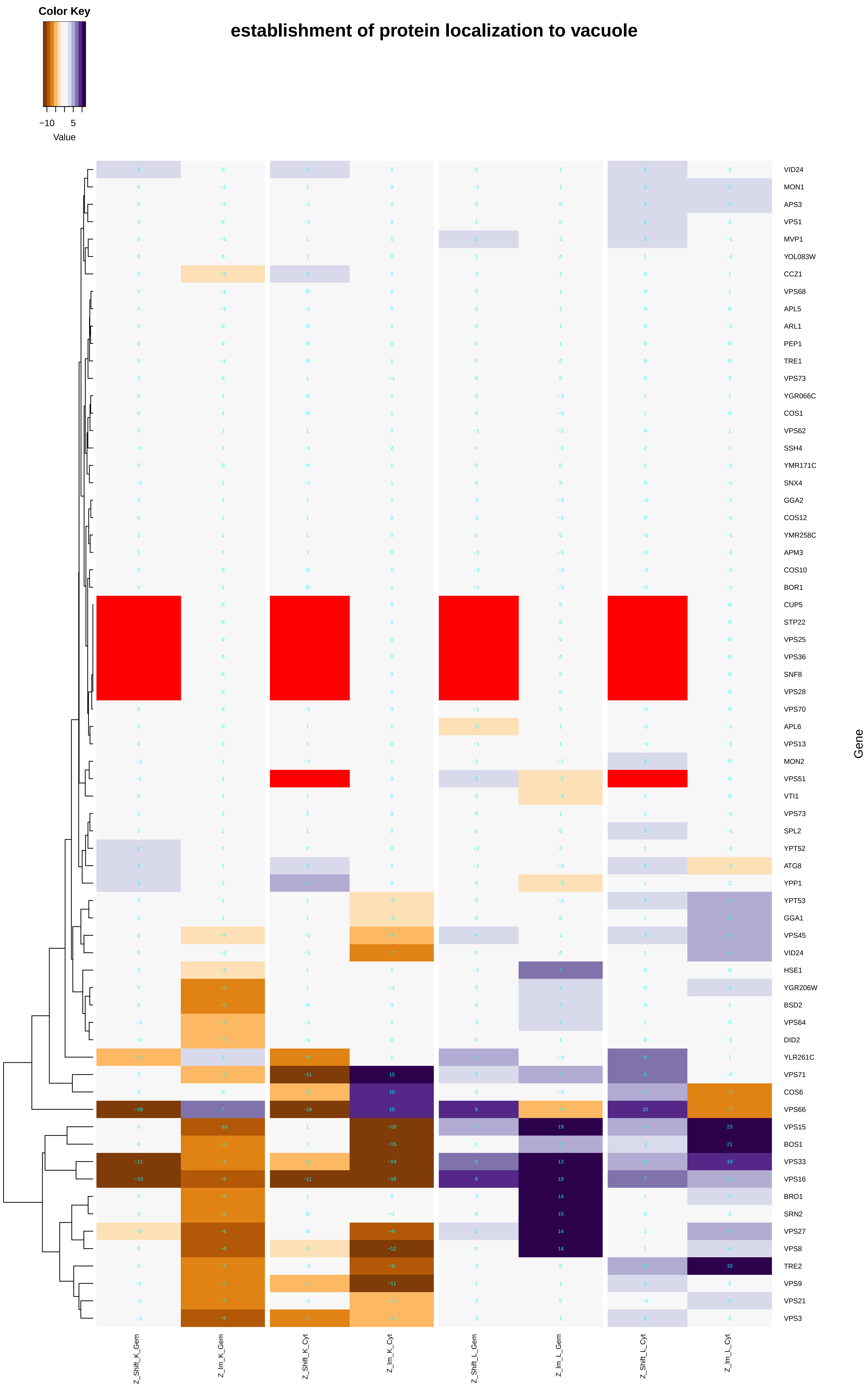
establishment of protein localization to vacuole
Color Key
−10
5
Value
Gene
VID24
MON1
APS3
VPS1
MVP1
YOL083W
CCZ1
VPS68
APL5
ARL1
PEP1
TRE1
VPS73
YGR066C
COS1
VPS62
SSH4
YMR171C
SNX4
GGA2
COS12
YMR258C
APM3
COS10
BOR1
CUP5
STP22
VPS25
VPS36
SNF8
VPS28
VPS70
APL6
VPS13
MON2
VPS51
VTI1
VPS73
SPL2
YPT52
ATG8
YPP1
YPT53
GGA1
VPS45
VID24
HSE1
YGR206W
BSD2
VPS64
DID2
YLR261C
VPS71
COS6
VPS66
VPS15
BOS1
VPS33
VPS16
BRO1
SRN2
VPS27
VPS8
TRE2
VPS9
VPS21
VPS3
Z_Shift_K_Gem
Z_Im_K_Gem
Z_Shift_K_Cyt
Z_Im_K_Cyt
Z_Shift_L_Gem
Z_Im_L_Gem
Z_Shift_L_Cyt
Z_Im_L_Cyt

protein localization to vacuolar membrane

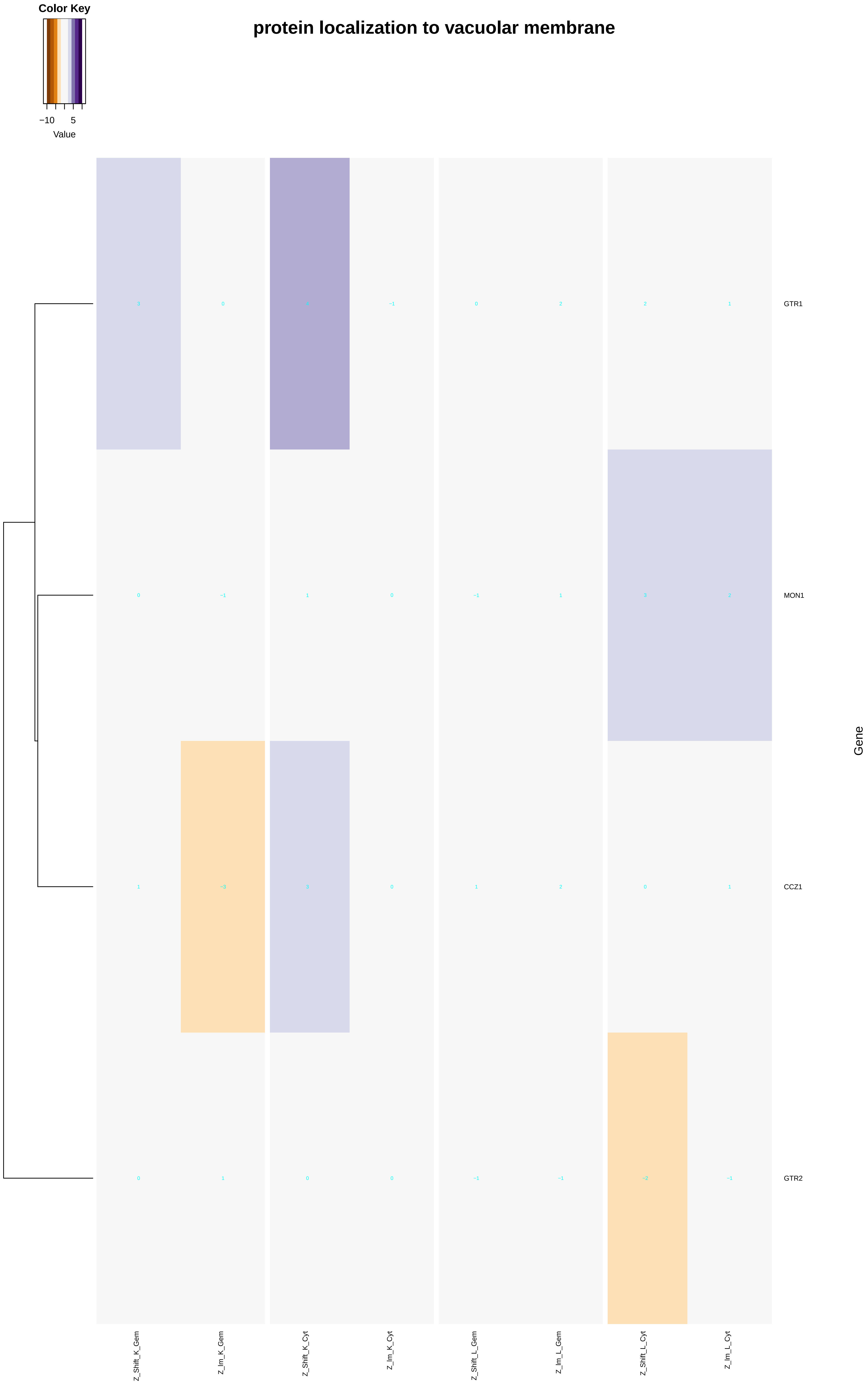

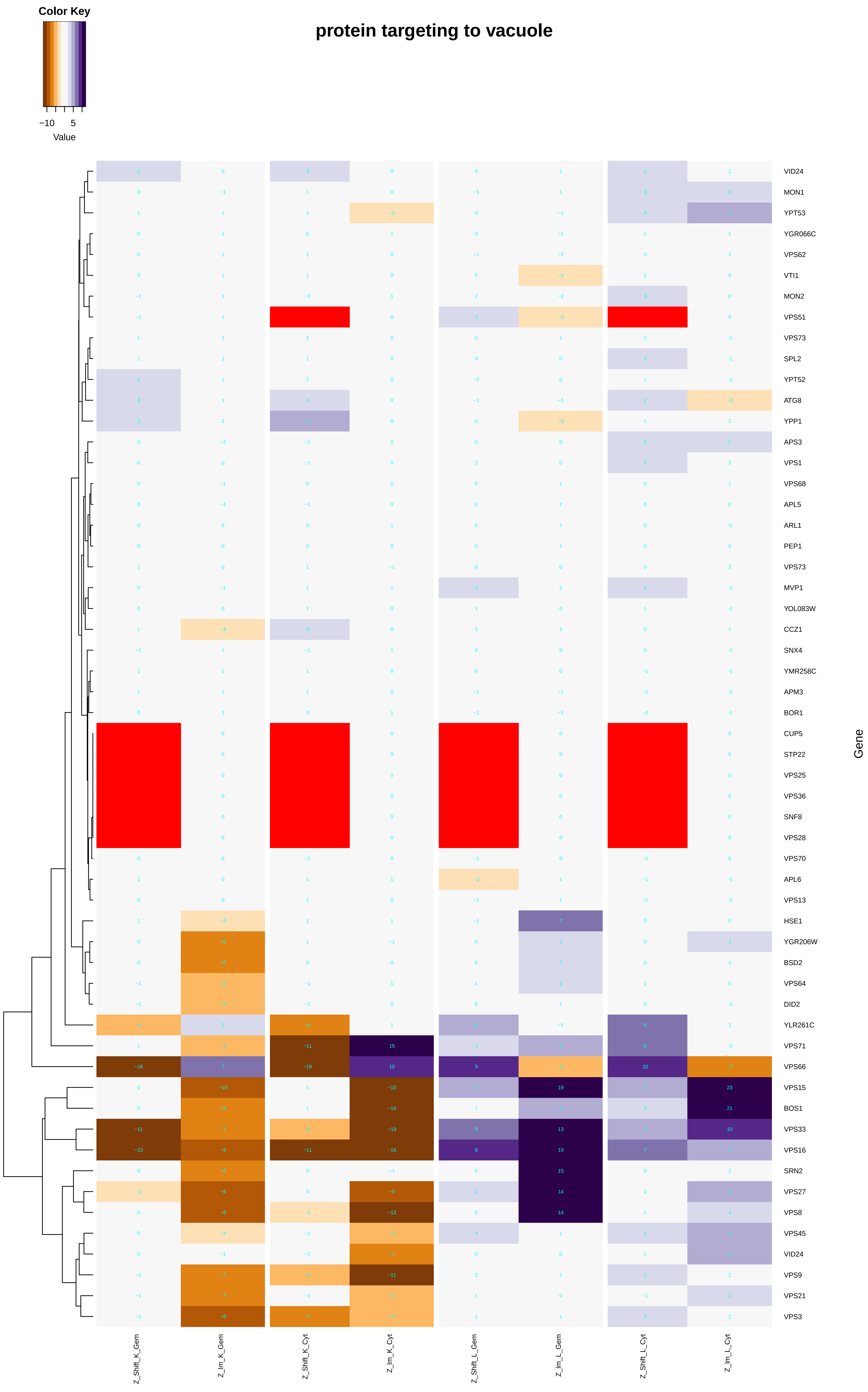

protein targeting to vacuole
Color Key
−10
5
Value
VID24
MON1
YPT53
YGR066C
VPS62
VTI1
MON2
VPS51
VPS73
SPL2
YPT52
ATG8
YPP1
APS3
VPS1
VPS68
APL5
ARL1
PEP1
VPS73
MVP1
YOL083W
CCZ1
SNX4
YMR258C
APM3
BOR1
CUP5
STP22
VPS25
VPS36
SNF8
VPS28
VPS70
APL6
VPS13
HSE1
YGR206W
BSD2
VPS64
DID2
YLR261C
VPS71
VPS66
VPS15
BOS1
VPS33
VPS16
SRN2
VPS27
VPS8
VPS45
VID24
VPS9
VPS21
VPS3
Gene
Z_Shift_K_Gem
Z_Im_K_Gem
Z_Shift_K_Cyt
Z_Im_K_Cyt
Z_Shift_L_Gem
Z_Im_L_Gem
Z_Shift_L_Cyt
Z_Im_L_Cyt

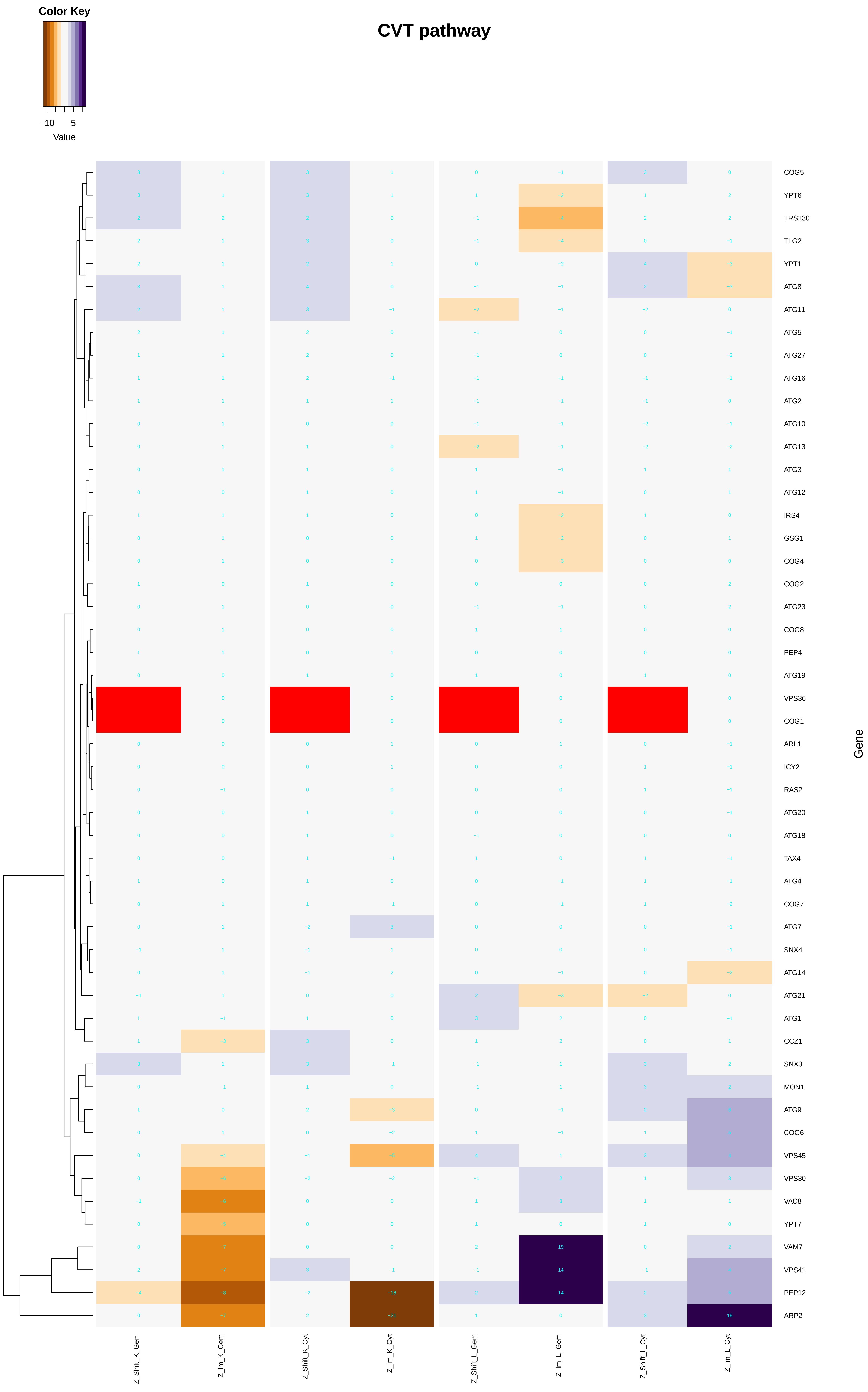
CVT pathway
Color Key
−10
5
Value
COG5
YPT6
TRS130
TLG2
YPT1
ATG8
ATG11
ATG5
ATG27
ATG16
ATG2
ATG10
ATG13
ATG3
ATG12
IRS4
GSG1
COG4
COG2
ATG23
COG8
PEP4
ATG19
VPS36
COG1
ARL1
ICY2
RAS2
ATG20
ATG18
TAX4
ATG4
COG7
ATG7
SNX4
ATG14
ATG21
ATG1
CCZ1
SNX3
MON1
ATG9
COG6
VPS45
VPS30
VAC8
YPT7
VAM7
VPS41
PEP12
ARP2
Gene
Z_Shift_K_Gem
Z_Im_K_Gem
Z_Shift_K_Cyt
Z_Im_K_Cyt
Z_Shift_L_Gem
Z_Im_L_Gem
Z_Shift_L_Cyt
Z_Im_L_Cyt

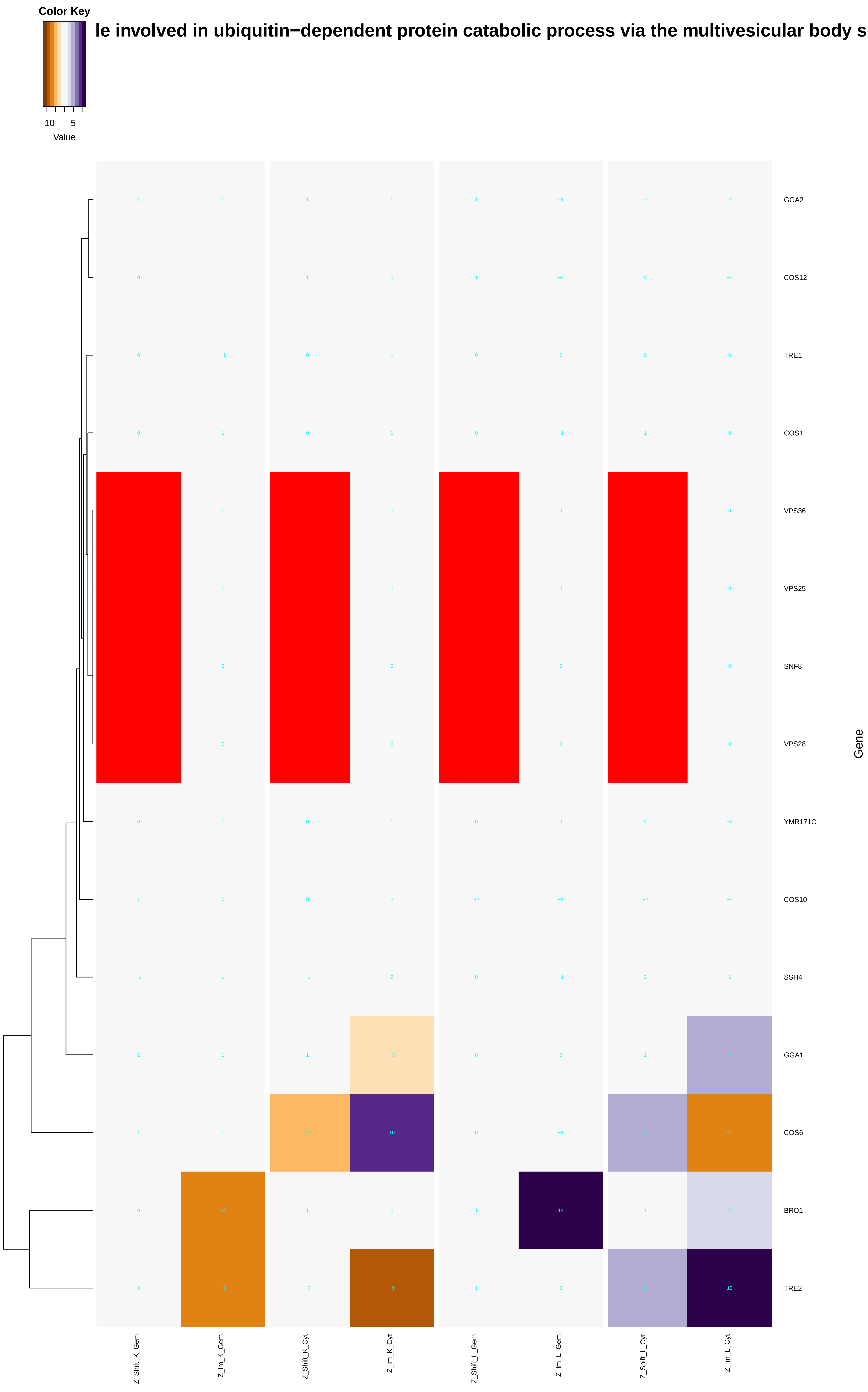
Color Key
−10 5
Value
le involved in ubiquitin−dependent protein catabolic process via the multivesicular body s
GGA2
COS12
TRE1
COS1
VPS36
VPS25
SNF8
VPS28
YMR171C
COS10
SSH4
GGA1
COS6
BRO1
TRE2
Gene
Z_Shift_K_Gem
Z_Im_K_Gem
Z_Shift_K_Cyt
Z_Im_K_Cyt
Z_Shift_L_Gem
Z_Im_L_Gem
Z_Shift_L_Cyt
Z_Im_L_Cyt
